# Clinical characterisation, treatment outcomes, and case fatality risk of patients with different SARS-CoV-2 variants in Bangladesh

**DOI:** 10.7189/jogh.14.05009

**Published:** 2024-06-21

**Authors:** Farzana Afroze, Mst Noorjahan Begum, Tahmeed Ahmed, Shams El Arifeen, Mohammed Ziaur Rahman, Ahmed Ehsanur Rahman, Mustafa Mahfuz, Md Farhad Kabir, Ahmedul Kabir, Robed Amin, Mohammad Shehab Uddin, Muhammad Asaduzzaman, Mohammad Abul Hasnat, Khairul Islam, Mohiuddin Sharif, Rezaul Hossain, Yasmin Jahan, Mustafizur Rahman, Mohammod Jobayer Chisti

**Affiliations:** 1 Nutrition Research Division, International Centre for Diarrhoeal Disease Research, Bangladesh (icddr,b), Dhaka, Bangladesh; 2 Infectious Diseases Division, icddr,b, Dhaka, Bangladesh; 3 Maternal and Child Health Division, icddr,b, Dhaka, Bangladesh; 4 Enteric and Respiratory Infection, Infectious Diseases Division, icddr,b, Dhaka, Bangladesh; 5 Administration, Directorate General of Health Services, Dhaka, Bangladesh; 6 Department of Non-Communicable Disease Control Program, Directorate General of Health Services, Dhaka, Bangladesh; 7 Kuwait Bangladesh Friendship Government Hospital, Dhaka, Bangladesh; 8 Department of Critical Care Medicine, Kuwait Bangladesh Friendship Government Hospital, Dhaka, Bangladesh; 9 Cardiology Department, Kurmitola General Hospital, Dhaka, Bangladesh; 10 Medicine Department, Dhaka medical college hospital, Dhaka, Bangladesh; 11 Department of Epidemiology, University of Washington, Seattle, USA; 12 Graduate School of Biomedical and Health Sciences, Hiroshima University, Japan

## Abstract

**Background:**

Bangladesh underwent four waves of the coronavirus disease 2019 (COVID-19) pandemic. Analysing them is essential for understanding changes in viral behaviour, disease patterns, severity, and response to treatment. Nevertheless, data are scarce in low- and middle-income countries. Therefore, we aimed to compare clinical manifestations; outcomes for therapy with oxygen, dexamethasone, and remdesivir; as well as the case fatality during the ancestral, alpha/beta, delta, and omicron-driven waves.

**Methods:**

We conducted an observational study at five hospitals in Dhaka, Bangladesh, with at least one dedicated COVID-19 unit for treating patients that followed national guidelines between November 2020 and February 2022. We collected data prospectively between 1 July 2021 and 30 September 2021 (delta) and retrospectively from 1 November 2020 to 4 March 2021 (ancestral), 5 March 2021 to 30 May 2021 (alpha/beta), and 1 January 2022 to 28 February 2022 (omicron), with the periods representing distinct waves of COVID-19. The primary outcome was 30-day case fatality across the waves. We used multivariable robust Poisson regression models with robust variance to estimate the 30-day case fatality risk ratio during various waves.

**Results:**

Among 966 participants, the rate of 30-day case fatality was comparable across different variants. However, the proportions of patients with fever (*P* < 0.001), cough (*P* < 0.001), breathing difficulty (*P* < 0.001), nausea (*P* < 0.001), fatigue (*P* < 0.001), headache (*P* < 0.001), diarrhoea (*P* < 0.001), loss of smell (*P* < 0.001), runny nose (*P* < 0.001), and chest pain (*P* = 0.001) were smaller during the omicron wave than the other three waves. After adjusting for potential confounders, the multivariable model showed that the likelihood of case fatality was significantly associated with age (adjusted risk ratio (aRR) = 1.05; 95% confidence interval (CI) = 1.04–1.07); hypoxaemia (aRR = 5.29; 95% CI = 1.58–17.7); critical disease (aRR = 6.45; 95% CI = 1.89–21.99), and modified early warning score ≥4 (aRR = 2.58; 95% CI = 1.71–3.88). We observed an 85% (aRR = 0.15; 95% CI = 0.03–0.72) reduction in case fatality among patients with any oxygen (L/min) compared to those without oxygen. However, individuals requiring ≥15 L/min of oxygen showed a significantly higher case fatality compared to those needing <15 L/min oxygen (aRR = 5.63; 95% CI = 2.68–11.81 for ancestral variant, aRR = 2.83; 95% CI = 1.25–6.41 for alpha/beta variant, aRR = 2.73; 95% CI = 1.56–4.77 for delta variant, aRR = 2.84; 95% CI = 1.56–5.16 for omicron variant). Remdesivir was associated with an increased case fatality during alpha/beta (aRR = 6.96; 95% CI = 1.54–31.43), delta (aRR = 4.13; 95% CI = 1.17–14.58), and omicron waves (aRR = 8.89; 95% CI = 2.46–32.13) compared to the ancestral wave. Dexamethasone administered during admission did not have any significant association with death (*P* = 0.239) in the entire cohort. However, dexamethasone reduced case fatality by 78% among the moderate to severe COVID-19 subgroup. We observed a 37% reduction in case fatality among vaccinated participants compared to those without vaccination (aRR = 0.63; 95% CI = 0.40–0.99).

**Conclusions:**

Our study provides insights into the clinical patterns, treatment impact, and case fatality across various SARS-CoV-2 variants in resource-limited settings. The findings underscored the crucial role of oxygen therapy and vaccination in reducing COVID-19 case fatality. They also emphasise the necessity for continuous disease surveillance and highlight the importance of close monitoring of patients with higher oxygen requirements (≥15 L/min) due to their association with fatal outcomes, as well as the significance of sustaining vaccination efforts and the need for clinical trials of newer antivirals in the ongoing battle against COVID-19.

The coronavirus disease 2019 (COVID-19) pandemic, caused by severe acute respiratory syndrome coronavirus 2 (SARS-CoV-2), has affected millions of people globally, resulting in more than six million deaths as of 18 October 2023. Although it is no longer classified as a global health emergency, the disease still poses a significant threat. Recent reports indicate that there were over 1.4 million new COVID-19 cases and over 1800 deaths between 31 July and 27 August 2023 worldwide [1].

Multiple variants of SARS-CoV-2 had emerged during the pandemic, and their dynamic behaviours continue to present unique challenges for health care systems. For example, the alpha, beta, gamma, delta, and omicron variants have led to surges in COVID-19 cases worldwide [2]. These variants, characterised by different mutations in the spike protein, are known as variants of concern. The alpha and beta variants, initially identified in the UK and South Africa, respectively, had a nearly 50% higher transmissibility than the original strain of the virus [3]. The gamma variant (first detected in Brazil), the delta variant (initially identified in India), and the omicron variant (emerging in South Africa) all had a greater number of mutations compared to the original strain. These mutations enhance their transmissibility, infectivity, and lead to variable clinical presentations, reduced treatment effectiveness, and variable mortality rates [4–7].

Since the first identification of COVID-19 cases on 8 March 2020, Bangladesh has experienced successive waves of infection, each linked to distinct variants, as shown in **Figure 1** [1,8]. The first wave, attributed to the ancestral strain, accounted for the majority of cases during the initial year and gradually waned by early March [9]. The second wave, dominated by the alpha and beta variants, emerged in March 2021 and lasted until May 2021 [10] (**Figure 2**). By June 2021, the Delta variant became dominant, with the number of reported cases peaking in July. The wave subsided, reaching its nadir in September 2021 [11]. Subsequently, the Omicron variant emerged in December 2021, driving the fourth wave and swiftly becoming the predominant variant, until this wave reached its nadir in March 2022 (**Figure 1**, **Figure 2**) [12]. These variants caused more than two million confirmed SARS-CoV-2 infections and 29 477 deaths as of 17 October 2023. However, these statistics do not reflect the true circumstances in low- and middle-income countries (LMICs) such as Bangladesh, as they faced challenges with SARS-CoV-2 testing [13], as well as those arising from factors such as population density, higher rates of non-communicable diseases, health care infrastructure limitations, and restricted access to advanced medical care [14,15]. Therefore, understanding the evolving nature of variants in LMICs would contribute to the global knowledge on the pandemic, but would also improve health care practices, resource allocation, and pandemic preparedness in these regions.

**Figure 1:**
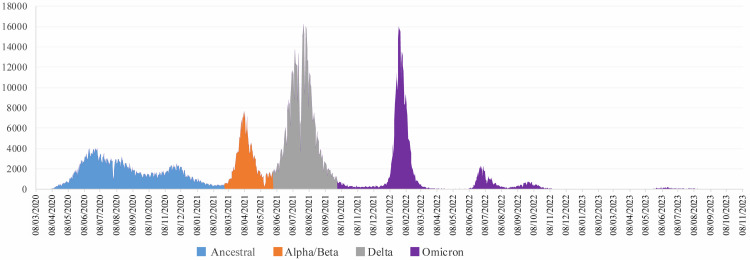
Four distinct waves of COVID-19 in Bangladesh: official daily reported cases during the pandemic.

**Figure 2:**
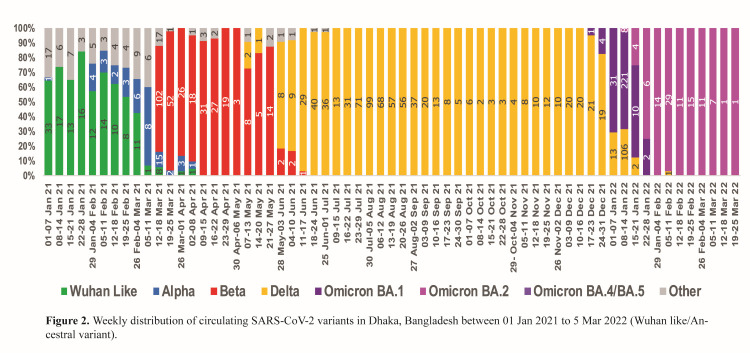
Weekly distribution of circulating SARS-CoV-2 variants between 1 January 2021 to 25 March 2022 in Dhaka, Bangladesh.

Since the beginning of the pandemic, oxygen therapy has been a mainstay in the treatment of hypoxaemia [16]. However, many therapeutic drugs were also tested for their potential to reduce COVID-19 severity and mortality, and management guidelines have evolved with findings from more comprehensive clinical trials. Thus, corticosteroid has become the standard of care for managing severe cases requiring oxygen therapy [17,18]. Similarly, remdesivir, a nucleoside analogue prodrug, showed favourable results in reducing mortality and risk of progression to invasive ventilation among severe and critical COVID-19 cases [19,20].

However, the mutational capacity of SARS-CoV-2 increases the probability that emerging variants of concern will continue to shape the future course of the pandemic, as they may bring a risk of altered therapeutic response or be vulnerable to existing drugs. However, data concerning the outcomes of patients infected with the predominant SARS-CoV-2 variants and treated with oxygen, dexamethasone, and remdesivir are limited, particularly in LMICs. Moreover, the consequence of the recurrent surges of various variants on the clinical presentations and outcomes of hospitalised COVID-19 patients in LMICs have largely remained unexplored. Therefore, understanding the impact of emerging variants on different treatment strategies is critical for mitigating health care disparities; optimising the allocation of essential resources such as intensive care unit (ICU) beds and oxygen supply; and safeguarding vulnerable populations in resource-constrained settings.

Therefore, we aimed to compare the clinical manifestations, outcomes of treatment in hospitalised COVID-19 patients during the ancestral, alpha/beta, delta and omicron-driven waves. Our findings would provide insights into the clinical patterns and treatment responses of SARS-CoV-2 variants, presenting a key step in addressing the challenges they pose in LMICs.

## METHODS

### Study design

This was a longitudinal observational study carried out in two different phases. We followed participants infected during delta dominated wave (1 July 2021 to 30 September 2021) prospectively for their 30-day fatality and extracted data on the same outcome from the medical records of participants who were infected during the ancestral (1 November 2020 to 4 March 2021), alpha/beta (5 March 2021 to 30 May 2021), and omicron (1 January 2022 to 28 February 2022) dominated waves. We included five tertiary hospitals for the prospective part and three hospitals for the retrospective part in Dhaka, the capital of Bangladesh. We selected these hospitals because they had at least one dedicated unit where COVID-19 patients were managed following national guidelines [21,22]. We included patients aged ≥18 years who tested positive for SARS-CoV-2 (either by reverse transcription polymerase chain reaction (RT-PCR) or rapid antigen test) and were admitted into the designated study hospitals with the moderate, severe or critical disease according to the classification of the World Health Organization (WHO) and the Bangladesh national guideline on COVID-19 case management [21,22].

### Data collection

For the prospective part, we enrolled consecutive consenting eligible participants within 48 hours of hospitalisation after initial stabilisation. For the retrospective component, we identified eligible COVID-19 patients from the hospital patient registry who were admitted between November 2020 and May 2021, as well as January and February 2021. Based on disease severity, all patients received standard management by the hospital physicians according to the WHO and Bangladesh national guidelines [21,22]. Hypoxaemic patients received oxygen at a rate of 2–10 L/min using nasal prongs or face masks and 10–14 L/min using non-rebreather oxygen masks. If the oxygen requirement increased to ≥15 L/min, non-invasive strategies, including high-flow oxygen therapy using high-flow nasal cannulas (HFNC) and non-invasive positive pressure ventilation, were applied before proceeding to intubation and invasive mechanical ventilation.

In the study hospitals, dexamethasone (6 mg/d for 10 days) was the standard of care for all COVID-19 patients with hypoxaemia. However, remdesivir was used at the discretion of the consultant working in the hospital. Patients also received other supportive management, including thromboprophylaxis with low molecular weight heparin or antibiotic therapy if a bacterial infection was confirmed or suspected. All enrolled patients were followed up daily for one month after enrolment in the hospital. The study staff collected the epidemiological data (including COVID-19 vaccination status); clinical, laboratory, and radiological features; treatment; and outcomes from patients’ hospital records and translated them into standardised study case records. Vital signs (heart rate (beats/min), systolic blood pressure (mm of Hg), respiratory rate (breaths/min), and temperature (°C)) and the level of consciousness using the alert, voice, pain, and unresponsive scale were assessed during admission. We then used these to calculate the modified early warning score (MEWS), which ranges from 0 to a maximum of 14, with higher scores indicating increased disease severity [23,24].

### Challenges and quality control

We faced challenges with data collection during the pandemic due to government measures, logistical constraints, and pandemic-related anxiety, as well as multiple lockdowns restricting movements and gatherings during the study period. Effectively managing the risk of infection among personnel also posed a significant challenge, which was addressed through rigorous training in infection prevention, control measures, and the proper use of personal protective equipment. Study physicians underwent comprehensive training on protocols, ensuring strict adherence to standardised procedures. We further held weekly virtual meetings involving study investigators, data collectors, statisticians, and other personnel to ensure data quality, address challenges encountered during data collection, and alleviate stress and anxiety associated with the ongoing pandemic.

For data management, we used the Research Electronic Data Capture (REDCap) software, a secure browser-based tool. Access to the electronic database and hard copies of case report forms was restricted to authorised study personnel. The study team, along with investigators, managed the database through a secure, password-protected process. The data management team performed quality control checks to ensure accuracy and completeness of the data. To further ensure data quality, study investigators reviewed 10% of all case report forms.

### SARS-CoV-2 variants and waves

Since January 2021, the International Centre for Diarrhoeal Disease Research, Bangladesh (icddr,b) study site has monitored SARS-CoV-2 variants in Bangladesh and collected 115 227 samples from suspected COVID-19 patients in Dhaka between 1 January 2021 and 31 March 2022 via various collection booths as part of the national testing system. The specimens were tested daily for the SARS-CoV-2 RNA using real-time PCR. Within this period, 24% (n = 27 697) of the suspected patients were positive for COVID-19. A subset of real-time PCR positive samples (n = 1259) with cycle thresholds (CT) values <30 were selected for SARS-CoV-2 variant screening using different sequencing platforms: sequencing spike gene by Sanger method; complete genome sequencing by Oxford Nanopore (MinION); and complete genome sequencing by Illumina (MiSeq) [25]. A wave of a distinct SARS-CoV-2 variant was defined as corresponding peaks in SARS-CoV-2 isolation, during which the isolated variant accounted for ≥70% of sequenced isolates in Dhaka. Patients diagnosed during the wave of a distinct variant were presumed to be infected with the prevailing variant (**Figure 2**).

### Outcome measures

Our primary endpoint was to compare the 30-day (from the enrolment time point) case fatality rates between the SARS-CoV-2-dominated waves. Our secondary outcomes of interest were the factors associated with the case fatality; the outcomes of patients affected with SARS-CoV-2 variants and treated with oxygen (L/min), remdesivir, and dexamethasone; factors associated with length of hospital stay in COVID-19 survivors; and a comparison of clinical presentation and treatment outcomes between waves.

### Sample size calculation

Based on the findings of an existing study, we assumed that the case fatality rate among moderate to severe or critical COVID-19 patients infected by the ancestral variant would be 14% [26]. Therefore, assuming 80% power, a 5% level of significance, and a desired margin of error of 0.05, we determined that 185 participants would be required in each group. Thus, accounting for a 10% dropout rate, at least a total of 815 patients needed to be enrolled for this study.

### Statistical analysis

We described continuous variables as medians and interquartile ranges (IQRs) and categorical variables as counts and percentages. To compare the general characteristics, clinical presentation, and treatment during hospitalisation in the various waves, we used the Kruskal-Wallis Rank Sum test for continuous variables and the χ^2^ test for categorical variables. Most of the explanatory variables had a low rate of missing values (below 2%). Specifically, age, sex, severity of COVID-19, prevalent variant, remdesivir, and dexamethasone had no missing values, while the variables of hypertension and supplemental oxygen had minimal missing values at 0.9% and 1.7%, respectively. Notably, there were no missing values observed for the primary and secondary outcomes.

As data collection during the pandemic was challenging, some data was missing. For example, the variables required to estimate MEWS (such as heart rate, systolic blood pressure, respiratory rate, temperature, and the alert, voice, pain, and unresponsive scale) had more than 5% of missing data. We therefore used multivariate imputation by chained equation method to impute these missing values. The complete variables included in the process were age, sex, disease severity, prevalent variant, and death. We conducted 10 cycles to obtain the final imputed data set, which we subsequently used to calculate the MEWS.

We followed the participants in our cohort for 30 days from enrolment and observed a case fatality rate of >10%; this means that the odds ratio could overemphasise the association between death and the explanatory variables. Therefore, we employed multivariable robust Poisson regression models with robust variance to estimate the risk ratio of the 30-day case fatality outcome for patients infected with the ancestral variant compared to patients infected with the alpha/beta, delta, and omicron variants. The selection of independent variables was guided by biological plausibility, an extensive literature review, and a univariate analysis with *P*-values <0.20. We included clinically relevant variables (including age, sex, hypoxemia, hypertension, disease severity, MEWS, and ICU requirement) in the model to control for their potential confounding effects.

To assess the effectiveness of treatments such as oxygen, remdesivir, and dexamethasone across different variants, we used SARS-CoV-2 variants as an interaction term with these treatments. This interaction term captured the heterogeneity of treatment effects, demonstrating how the impact of specific treatments (oxygen, dexamethasone, and remdesivir) on death varies across prevalent SARS-CoV-2 variants (ancestral, alpha/beta, delta, and omicron). Thus, we built three models where death was the dependent variable; age, sex, disease severity, MEWS, ICU requirement, prevalent SARS-CoV-2 variant, oxygen therapy, remdesivir, dexamethasone, hypoxemia, and hypertension were the independent variables; and the prevalent variant with oxygen ≥15L/min (model 1), dexamethasone (model 2), and remdesivir (model 3) were the interaction terms. We applied similar models to estimate the factors associated with the median hospital stay (≤8 days) across different SARS-CoV-2 variants, where the median days for the entire sample established the hospital stay cutoff. We then estimated the risk ratios and the corresponding 95% confidence intervals (CIs), and set the significance level at 5%, with a *P*-value <0.05 denoting statistical significance.

To investigate the association between case fatality and treatment with dexamethasone and remdesivir among moderate to severe COVID-19 cases (n = 473), we conducted a subgroup analysis to determine whether these treatments might benefit such cases. The subgroup analysis was not predetermined. We used multivariable robust Poisson regression models to assess the association between fatality and treatment in moderate to severe COVID-19. However, this analysis suffers from insufficient power due to a smaller sample size. We used Stata, version 15.0 (StataCorp LLC, College Station, Texas, USA) for statistical analyses.

## RESULTS

### Patient characteristics

We enrolled 966 participants between November 2020 and February 2022; 399 patients from five hospitals were included prospectively during the delta dominated period and 567 patients from three hospitals were included retrospectively during the ancestral, alpha/beta, and omicron dominated periods. The participants had a median age of 55 years (IQR = 45–65). The proportion of male participants were significantly different between the subgroups (*P* = 0.001), being higher in ancestral (63.8%) than in the delta (47.4%) and omicron (52.7%) waves (**Table 1**).

**Table 1:** Sociodemographic, clinical and laboratory characteristics by prevalent SARS CoV-2 variant among COVID-19 patients in Dhaka, Bangladesh*

	Total (n = 966)	Ancestral variant (n = 199)	Alpha/beta (n = 144)	Delta (n = 399)	Omicron (n = 224)	*P*-value
**Characteristics**						
Age	55 (45–65)	56 (47–65)	54 (44–64)	55 (45–65)	54 (41–66.5)	0.572
Male, n (%)	520 (53.8)	127 (63.8)†	86 (59.7)‡	189 (47.4)	118 (52.7)	0.001
COVID-19 vaccination, n/N (%)	237/860 (27.6)	NA	16/90 (17.8)	69/396 (17.4)	152/214 (71.0)§	<0.001
Time since COVID-19 diagnosis to admission in days	1 (0–3)	1 (0–3)	2 (1–4)	1 (1–4)	0 (0–1)‖	<0.001
Time since symptom onset to admission in days	7 (5–10)	7 (4–10)	7 (5–10)	8 (6–10)¶	4 (1–7)‖	<0.001
**Symptoms, n/N (%)**						
Fever	708/924 (76.6)	146/191 (76.4)§	114/131 (87.0)‡	361/378 (95.5)	87/224 (38.8)‖	<0.001
Cough	646/878 (73.6)	143/171 (83.6)	106/120 (88.3)	317/363 (87.3)	80/224 (35.7)‖	<0.001
Sore throat	79/682 (11.6)	17/105 (16.2)	14/81 (17.3)	36/272 (13.2)	12/224 (5.4)‖	0.003
Headache	122/688 (17.7)	36/111 (32.4)	25/82 (30.5)	42/271 (15.5)¶	19/224 (8.5)‖	<0.001
Diarrhoea	111/689 (16.1)	24/107 (22.4)	24/83 (28.9)	56/275 (20.4)	7/224 (3.1)‖	<0.001
Breathing difficulty	502/835 (60.1)	97/153 (63.4)‡	76/108 (70.4)	273/350 (78.0)	56/224 (25.0)‖	<0.001
Chest pain	39/663 (5.9)	5/95 (5.3)	7/73 (9.6)	25/271 (9.2)	2/224 (0.9)‖	0.001
Loss of smell	86/675 (12.7)	1/92 (1.1)‡	3/68 (4.4)	82/291 (28.2)	0/224 (0.0)§	<0.001
Runny nose	76/680 (11.2)	21/103 (20.4)	11/76 (14.5)	39/277 (14.1)	5/224 (2.2)‖	<0.001
Nausea	160/712 (22.5)	44/119 (37.0)	31/89 (34.8)	68/280 (24.3)¶	17/224 (7.6)‖	<0.001
Fatigue	142/689 (20.6)	14/100 (14.0)§	22/80 (27.5)	98/285 (34.4)	8/224 (3.6)‖	<0.001
**Physical findings**						
Respiratory rate	29 (24–36)	26 (20–32)	28 (22–40)	30 (24–36)**	22 (20–26)	0.001
Hypoxaemia, n/N (%)	532/958 (55.5)	69/199 (34.7)††	65/140 (46.4)	290/398 (72.9)***	108/221 (48.9)	<0.001
Pulse rate	92 (83–105)	92.5 (84–104)	95.5(83.5–107.5)	91 (80–103)	94 (84–106)	0.126
Systolic blood pressure	125 (112–138)	125 (111–137)	125 (111–138)	123 (111–133)	130 (120–148)¶	0.008
Diastolic blood pressure	79 (70–86)	79 (70–85)	80 (70–88)	78 (70–85)	80 (69–91)	0.216
**Disease severity, n (%)**						
Moderate	306 (31.7)	91 (45.7)	53 (36.8)	71 (17.8) ‖	91 (40.6)	<0.001
Severe	167 (17.3)	30 (15.1)	16 (11.1)	62 (15.5)	59 (26.3)‖	<0.001
Critical	493 (51.0)	78 (39.2)§	75 (52.1)	266 (66.7)‡‡	74 (33.0)§	<0.001
MEWS≥4	386 (40.0)	73 (36.7)	69 (47.9)	163 (40.9)	81 (36.2)	0.104
**Comorbidities, n/N (%)**						
Diabetes mellitus	489/958 (51.0)	95/198 (48.0)	78/138 (56.5)	211/398 (53.0)	105/224 (46.9)	0.205
Hypertension	555/957 (58.0)	113/198 (57.1)	87/137 (63.5)	215/398 (54.0)	140/224 (62.5)	0.101
COPD/asthma	165/953 (17.3)	36/198 (18.2)	27/135 (20.0)	62/396 (15.7)	40/224 (17.9)	0.662
Ischaemic heart disease	169/954 (17.7)	36/197 (18.3)	21/135 (15.6)	71/398 (17.8)	41/224 (18.3)	0.912
Chronic liver disease	11/939 (1.2)	2/189 (1.1)	4/130 (3.1)	4/397 (1.0)	1/223 (0.4)	0.189
Hypothyroidism	56/936 (6.0)	13/186 (7.0)	6/131 (4.6)	23/396 (5.8)	14/223 (6.3)	0.838
Chronic kidney disease	115/951 (12.1)	17/193 (8.8)	9/136 (6.6)	39/398 (9.8)	50/224 (22.3)‖	<0.001
Immunocompromised conditions	22/938 (2.3)	4/188 (2.1)	1/132 (0.8)	9/394 (2.3)	8/224 (3.6)	0.397
**Laboratory findings**						
Any HRCT abnormalities during admission, n/N (%)	170/966 (17.6)	16/199 (8.0)§§	5/144 (3.5)	146/399 (36.6)‖‖	3/224 (1.3)	<0.001
Blood glucose in mmol/L	9.0 (6.7–13.6)	8.0 (6.8–12)	9.1 (6.8–13.6)	10.2 (7.2–15.4)¶¶	8.85 (5.65–13.9)	0.027
Haemoglobin in gm/dl, x̄ (SD)	11.9 (1.8)	12.2 (1.7)	12.3 (1.9)	11.8 (1.8)	11.5 (2.1)¶	0.004
White cell count in 10^9^/L	7.1 (5.2–9.5)	6.9 (5.2–9.36)	6.8 (5.3–9.0)	7.1 (5.1–9.4)	8.6 (6.3–10.8)	0.072
Platelet count in 10^9^/L	220 (171–283)	210 (170–278)	200 (162.5–252)	221 (168–279.5)	266 (203–347)‖	0.004
D-dimer in ng/ml	481 (276–950)	392 (189–673)	433(220–860)	520 (308–960)¶¶	570 (170–1510)	0.021
Fibrinogen in mg/dl, x̄ (SD)	462.7 (137.6)	469.5 (135)	473.1 (127.7)	426.7 (175)	NA	0.788
Ferritin in ng/ml	418 (175–767)	446 (238–764)	283 (158–506)	418 (173–880)	424 (146–681)	0.265
Sodium in mmol/L	136 (133–140)	136∙2 (134–139)	134 (132–137)	136 (133–139)	141 (135–145)‖	<0.001
Potassium in mmol/L	4.1 (3.7–4.6)	3.9 (3.6–4.1)	3.8 (3.5–4.3)†	4.2 (3.8–4.7)¶	4.2 (3.7–4.5)	<0.001
Chloride in mmol/L	100 (97–103)	101 (97–103)	100.13 (98–104)	100 (96–103)	102 (99–105)‡	0.028
Total CO_2_ in mmol/L	25 (22–27.2)	25 (23.9–27)	23.5 (21.1–26.2)	25.4 (22–28)	25 (25–25)	0.241
Creatinine in μmol/L	1.1 (0.9–1.4)	1.1 (1.0–1.4)	1.0 (0.9–1.3)	1.1 (0.9–1.4)	1.2 (1.0–2.1)	0.162
C-reactive protein in mg/L	24 (12–78)	28.0 (8.6–93.6)	31.4 (12.0–68.3)	24 (12–88)	15.0 (5–29.1)‖	0.036
Lactate dehydrogenase	426 (314–606)	299 (235–367)	435 (228–703)	544 (426–642)¶¶	435 (204–804)	<0.001

COPD – chronic obstructive pulmonary disease, MEWS – modified early warning score, NA – not applicable, x̄ – mean

*Presented as median (IQR) unless specified otherwise.

†Delta and omicron variants.

‡Delta variants.

§Alpha/beta and delta variants.

‖Alpha/beta, ancestral and delta variants.

¶Alpha/beta and ancestral variants.

**Ancestral and omicron variants.

††Alpha/beta, delta and omicron variants

‡‡Alpha/beta variants.

§§Omicron variant.

‖‖Alpha/beta, ancestral and omicron variants.

††Alpha/beta and delta variants.

¶¶Ancestral variants.

***Alpha/beta and omicron variants.

The two most common comorbidities were hypertension (n/N = 555/957, 58%) and diabetes (n/N = 489/958, 51%). The proportion of patients with chronic kidney disease was higher during the omicron wave (22.3%) than the other three waves (ancestral: 8.8%, alpha/beta: 6.6%, delta: 9.8%; *P* < 0.001). The symptoms on admission varied significantly within prevalent variants (**Table 1**). Fewer patients presented with fever during the omicron wave (38.8%) compared to the ancestral (76.4%), alpha/beta (87%), and delta (95.5%) waves (*P* < 0.001). We observed the same trend for cough (omicron: 35.7%, ancestral: 83.6%, alpha/beta: 88.3%, delta: 87.3%; *P* < 0.001), breathing difficulty (omicron: 25%, ancestral: 63.4%, alpha/beta: 70.4%, delta: 78%; *P* < 0.001), nausea (omicron: 7.6%, ancestral: 37%, alpha/beta: 34.8%, delta: 24.3%; *P* < 0.001), fatigue (omicron: 3.6%, ancestral: 14%, alpha/beta: 27.5%, delta: 34.4%; *P* < 0.001), headache (omicron: 8.5%, ancestral: 32.4%, alpha/beta: 30.5%, delta: 15.5%; *P* < 0.001), and other symptoms, including diarrhoea (*P* < 0.001), loss of smell (*P* < 0.001), runny nose (*P* < 0.001), and chest pain (*P* = 0.001).

During the delta wave, a higher proportion of patients presented with fever (95.5% vs 76.4%), breathing difficulty (78% vs 63%), loss of smell (28.2% vs 1.1%) and fatigue (34.4% vs 14%) than the ancestral wave (**Table 1**). Tachypnoea (80.9% vs 33% and 68%; *P* = 0.035) and hypoxaemia (72.9% vs 48.9% and 46.4%; *P* < 0.001) were more common during the delta wave than the omicron or alpha/beta wave. Moreover, 66.7% (n/N = 266/399) of patients during the delta wave had a diagnosis of critical COVID-19 compared to 39.2% (n/N = 78/199) and 33% (n/N = 74/224) during the ancestral and omicron wave, respectively (*P* < 0.001). However, 26.3% (n/N = 59/224) of patients had severe COVID-19 during omicron wave, significantly higher than the other three waves (*P* < 0.001). A total of 386/966 (40%) participants had a MEWS score ≥4 during hospitalisation. However, no statistically significant difference was observed between the groups (*P* = 0.104).

High-resolution computed tomography abnormalities were more often found during the delta wave than the other three waves (delta: 36.6%, ancestral: 8%, alpha/beta: 3.5%, omicron: 1.3%; *P* < 0.001). We found significant difference in blood glucose (*P* = 0.027), haemoglobin (*P* = 0.022), total white blood cells (*P* < 0.001), platelets (*P* = 0.004), d-dimer (*P* = 0.021), lactate dehydrogenase (*P* < 0.001), sodium (*P* < 0.001), potassium (*P* < 0.001), and chloride (*P* = 0.028) between the cohorts. More patients in the omicron wave (n/N = 152/214, 71%) received two or more doses of COVID-19 vaccine than in the ancestral (0%), alpha/beta (n/N = 16/90, 18%), or delta (n/N = 69/396, 17.4%,) waves.

### Clinical outcomes

**Figure 3:**
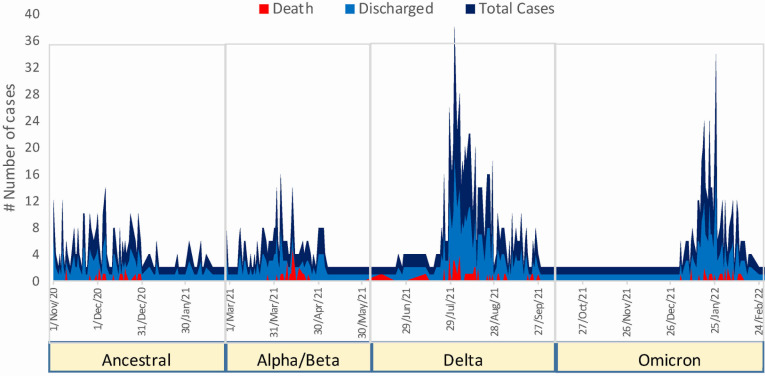
Distribution of 30-day inpatient deaths and enrolled cases during the ancestral, alpha/beta, delta, and omicron dominated waves in Dhaka, Bangladesh.

There were 9.6% (n/N = 19/199), 16% (n/N = 23/144), 11.3% (n/N = 45/399), and 10.7% (n/N = 24/224) deceased patients during the ancestral, alpha/beta, delta, and omicron wave, respectively (**Figure 3**, **Table 2**). A significantly higher proportion of patients left the hospital against medical advice during the omicron wave compared to other three waves (*P* < 0.001). The actual risk of fatality was 11 times higher (95% CI = 4.15–30.03, *P* < 0.001) among individuals over 60 years than those below 40 years of age. The absolute risks of case fatality for participant aged 18 to 40 years, 41 to 60 years, and above 60 years were 2.1%, 6.36%, and 23.51%, respectively. Compared to individuals with moderate COVID-19, the risk of fatality was 3.4 (95% CI = 1.38–8.37) and eight times (95% CI = 3.79–17.2) higher among individuals with severe and critical COVID-19, respectively (Table S1 in the **Online Supplementary Document**). We observed a 37% reduction in case fatality (aRR = 0.63; 95% CI = 0.40–0.99) among vaccinated participants compared to those without vaccination in the multivariable robust Poisson regression model, after adjusting for potential confounders such as age, hospital stay, no ICU requirement, and SpO_2_ (Table S2 in the **Online Supplementary Document**).

**Table 2:** Outcomes and oxygen therapy in hospitalised COVID-19 patients by prevalent SARS CoV-2 variants during the study period in Dhaka, Bangladesh*

	Total (n = 966)	Ancestral variant (n = 199)	Alpha/beta (n = 144)	Delta (n = 399)	Omicron (n = 224)	*P*-value
**Characteristics**						
Thirty-day case fatality, n (%)	111 (11.5)	19 (9.6)	23 (16.0)	45 (11.3)	24 (10.7)	0.292
Discharge, n (%)	754 (78.1)	174 (87.40)†	118 (81.9)	321 (80.5)	141 (63.0)‡	<0.001
Left against medical advice, n (%)	60 (6.2)	2 (1.01)	1 (0.7)	11 (2.76)	46 (20.5)‡	<0.001
Referred to other facilities, n (%)	41 (4.2)	4 (2.01)†	2 (1.4)†	22 (5.5)	13 (5.8)§	0.041
Required ICU care, n (%)	503/911 (55.2)	83/180 (46.1)‖	75/124 (60.5)	268/389 (68.9)	77/218 (35∙3)‡	<0.001
Hospital stay in days, MD (IQR)	8 (5–12)	10 (5–15)	8 (5–13)	8 (5–11)	6 (3–10)‡	<0.001
Median hospital stays (≤8 d), n (%)	536 (55.5)	83 (41.7)‖	79 (54.9)	223 (55.9)	151 (67.4)‡	<0.001
Any supplemental oxygen in L/min, n/N (%)	644/950(67.8)	103/193 (53.4)	80/138(58.0)	329/397(82.9)¶	132/222(59.5)	<0.001
**Oxygen therapy <15 L/min**						
n/N (%)	535/950 (56.3)	92/193 (47.7)	67/138 (48.6)	264/397 (66.5)¶	112/222 (50.5)	<0.001
Oxygen flow, MD (IQR)	5 (3–7)	4 (3–5)**	5 (4–7)	5 (3–8)	5 (4–5.5)	0.003
*Nasal cannula, n/N (%)*	323/535 (60.4)	76 (82.6)**	43 (64.2)	160 (60.6)	44 (39.3)‖	<0.001
*Face mask, n/N (%)*	172/535 (32.2)	15 (16.3)**	22 (32.8)	79 (29.9)	56 (50.0)‖	<0.001
*Non-rebreather mask, n/N (%)*	40/535 (7.5)	1 (1.1)††	2 (3.0)	25 (9.5)	12 (10.7)	0.014
**Oxygen therapy ≥15 L/min**						
n/N (%)	109/950 (11.5)	11/193 (5.7)	13/138 (9.4)	65/397 (16∙4)¶	20/222 (9.0)	0.001
Oxygen flow, MD (IQR)	15 (15–15)	15 (15–15)	15 (15–38)	15 (15–15)‡‡	15 (15–20)	0.010
*Face mask, n/N (%)*§§	16/109 (14.7)	0/11 (0)	0 (0)	16/65 (24∙6)¶	0/20 (0)	0.004
*Non-rebreather mask, n/N (%)*‖‖	70/109 (64.2)	6/11 (54.6)	9/13 (69.2)	45/65 (69.2)	10/20 (50.0)	0.374
*High-flow nasal cannula, n/N (%)*	15/109 (13.8)	2/11 (18.2)	4/13 (30.8)	4/65 (6.2)‡‡	5/20 (25.0)	0.016
Bilevel-positive airway pressure, *n/N (%)*	6/109 (5.5)	3/11 (27.3)†	0/13 (0)	0 (0)	3/20 (15.0)†	0.001
*Mechanical ventilation, n/N (%)*	2/109 (1.8)	0/11 (0)	0/13 (0)	0 (0)	2/20 (10.0)	0.079
**Duration of oxygen therapy in hours, MD (IQR)**						
Any supplemental oxygen	48 (24–120)	48 (24–96)	72 (24–120)	72 (24–168)	24 (24–48)‡	<0.001
High-flow nasal cannula	48 (24–120)	48 (24–48)	48 (24–120)	72 (48–144)	60 (13–96)	0.130
Bilevel-positive airway pressure	72 (24–144)	72 (24–96)	72 (48–96)	168 (24–219)	41 (24–108)	0.344
Mechanical ventilation	22 (14–42)	48 (48–48)	24 (24–24)	4 (4–4)	19 (17–36)	0.372

ICU – intensive care unit, IQR – interquartile range, MD – median, NA – not applicable

*Presented as n/N (%) unless specified otherwise.

†Delta variant.

‡Ancestral, alpha/beta, and delta variants.

§Alpha/beta and ancestral variants.

‖Alpha/beta and delta variants.

¶Ancestral, alpha/beta, and omicron variants.

**Alpha/beta, delta and omicron variants

††Omicron and delta variants.

‡‡Alpha/beta and omicron variants

§§Combination of nasal cannula and face mask.

‖‖Combination of nasal cannula and non-rebreather mask.

The median duration of hospital stay was 6 days (IQR = 3–10) during the omicron wave vs 10 (IQR = 5–15), 8 (IQR = 5–13), and 8 days (IQR = 5–11) during the ancestral, alpha/beta, and delta waves, respectively (*P* < 0.001). Overall, 67.8% (n/N = 644/950) of patients received any form of oxygen (L/min); 83% (n = 535/644) received <15 L/min, while 17% (n/N = 109/644) received ≥15¸L/min oxygen. Among 109 patients who required ≥15 L/min of oxygen, 70 (64.2%), 16 (14.7%), 15 (13.8%), 6 (5%), and 2 (1.8%) patients received oxygen using a combination of non-rebreather masks and nasal cannula; face mask and nasal cannula; HFNCs; bilevel positive airway pressure; and mechanical ventilation, respectively (**Table 2**). Moreover, 82.9% (n/N = 329/397) of patients during the delta wave required oxygen compared to 53.4% (n/N = 103/193), 58% (n/N = 80/138), and 59.5% (n/N = 132/222) during the ancestral, alpha/beta, and omicron waves, respectively (*P* < 0.001). The median duration of any form of oxygen (L/min) was 24 hours (IQR = 24–48) during the omicron wave, compared to 72 hours (IQR = 24–168) during the delta and alpha/beta (IQR = 24–120) and 48 (IQR = 24–96) hours during the ancestral wave (*P* < 0.001). Lastly, 77/218 (35.3%) of patients required ICU care during the omicron wave compared to 83/180 (46.1%), 75/124 (60.5%), and 268/389 (68.9%) during the ancestral, alpha/beta, and delta wave respectively (*P* < 0.001).

In view of treatments for the patients hospitalised during the prevalent SARS CoV-2 surges, we observed more frequent use of remdesivir during alpha/beta (34%, n/N = 49/144) and delta (42.6%, n/N = 170/399) wave than ancestral (16.1%, n/N = 32/199) and omicron (12.1%, n/N = 27/224) wave (*P* = 0.002) (**Table 3**). Ivermectin treatment decreased from 8% during the ancestral wave to 4.8% during delta and none during omicron wave (*P* < 0.001). Further, 521/966 (53.9%) of patients were treated with dexamethasone during hospitalisation, while 284/399 (71.2%) got dexamethasone during the delta wave vs 70/199 (35.2%),  74/144 (51.4%), and 93/224 (41.5%) during the ancestral, alpha/beta, and omicron waves, respectively (*P* < 0.001). A higher proportion of patients received anticoagulant therapy during the delta wave compared to the other waves (*P* < 0.001).

**Table 3:** Treatments during hospitalisation by prevalent SARS CoV-2 variants during the study period in Dhaka, Bangladesh, presented as n (%)

	Total (n = 966)	Ancestral variant (n = 199)	Alpha/beta (n = 144)	Delta (n = 399)	Omicron (n = 224)	*P*-value
**Antibiotics**	906 (93.8)	190 (95.5)	137 (95.1)	377 (94.5)	202 (90.2)	0.080
Ceftriaxone	604 (62.5)	116 (58.3)	90 (62.5)	259 (64.9)	139 (62.1)	0.473
Ciprofloxacin	68 (7.0)	30 (15.1)*	22 (15.3)*	15 (3.8)†	1 (0.4)	<0.001
Meropenem	162 (16.8)	33 (16.6)	26 (18.1)	71 (17.8)	32 (14.3)	0.690
Tigecycline	14 (1.4)	1 (0.5)	4 (2.8)	9 (2.3)	0 (0.0)	0.918
Others	478 (49.5)	116 (58.3)*	97 (67.4)*	167 (41.9)	98 (43.8)	<0.001
**Antivirals**	284 (29.4)	36 (18.1)‡	51 (35.4)	170 (42.6)	27 (12.1)‡	<0.001
Favipiravir	6 (0.6)	4 (2.0)§	2 (1.4)	NA	NA	0.009
Remdesivir	278 (28.8)	32 (16.1)	49 (34.0)‖	170 (42.6)‖	27 (12.1)	0.002
Ivermectin	46 (4.8)	16 (8.0)†	11 (7.6)†	19 (4.8)†	0 (0.0)	<0.001
**Steroids**	543 (56.2)	83 (41.7)	78 (54.2)‖	285(71.4)¶	97 (43.3)	<0.001
Methyl prednisolone	18 (1.9)	10 (5.0)*	2 (1.4)	4 (1.0)	2 (0.9)	0.008
Dexamethasone	521 (53.9)	70 (35.2)‡	74 (51.4)**	284 (71.2)†	93 (41.5)	<0.001
Hydrocortisone	10 (1.0)	4 (2.0)	2 (1.4)	2 (0.5)	2 (0.9)	0.365
**Anti-coagulant**	784 (81.2)	159 (79.9)†	115 (79.9)†	363(91.0)¶	147 (65.6)	<0.001
Enoxaparin	757 (78.4)	151 (75.9)†	106 (73.6)	354(88.7)¶	146 (65.2)	<0.001
Low molecular weight heparin	8 (0.8)	2 (1.0)	4 (2.8)**	1 (0.3)	1 (0.4)	0.033
Rivaroxaban	29 (3.0)	8 (4.0)	5 (3.5)	15 (3.8)	1 (0.4)	0.085
Other	1 (0.1)	NA	NA	1 (0.3)	NA	NA

NA – not applicable

*Delta and omicron variants.

†Omicron variant.

‡Alpha/beta and delta variants.

§Alpha/beta variants.

‖Ancestral and omicron variants.

¶Alpha/beta, ancestral, and omicron variants

**Delta variants.

### Impact of variants

The multivariable robust Poisson regression model showed a comparable case fatality between the ancestral, alpha/beta (*P* = 0.605), delta (*P* = 0.870), and omicron (*P* = 0.775) dominated waves (**Table 4**, model 2). The patients diagnosed with critical disease had a higher likelihood of death compared to those with moderate disease (aRR = 6.45; 95% CI = 1.89–21.99). Similarly, patients with a MEWS score ≥4 had a higher risk of fatality compared to those with MEWS<4 (aRR = 2.58; 95% CI = 1.71–3.88). A one-year increment in age conferred a 4% to 5% increase in case fatality (**Table 4**) in one month. COVID-19 patients who did not require ICU care had a 69% reduction in case fatality (aRR = 0.31; 95% CI = 0.12, 0.81) compared to those who required ICU care.

**Table 4:** Impact of treatment and risk of death in alpha/beta, delta, and omicron variant compared with ancestral variant in hospitalised COVID-19 patients

	Model 1*		Model 2†		Model 3‡		
	**aRR (95% CI)**	***P*-value**	**aRR (95% CI)**	***P*-value**	**aRR (95% CI)**	***P*-value**
**Characteristics**						
Male	1.2 (0.83–1.73)	0.342	1.51 (1–2.29)	0.049	1.44 (0.96–2.17)	0.077
Age in years	1.04 (1.03–1.06)	<0.001	1.05 (1.04–1.07)	<0.001	1.06 (1.04–1.07)	<0.001
Hypoxaemia	NA	NA	5.29 (1.58–17.7)	0.007	5.43 (1.65–17.82)	0.005
Hypertension	1.3 (0.9–1.87)	0.165	1.4 (0.92–2.11)	0.112	1.39 (0.93–2.09)	0.111
**Disease severity**						
Moderate	NA	NA	ref		ref	
Severe	NA	NA	2.86 (0.93–8.81)	0.068	2.64 (0.86–8.16)	0.091
Critical	NA	NA	6.45 (1.89–21.99)	0.003	5.6 (1.63–19.22)	0.006
MEWS≥4	1.80 (1.23–2.64)	0.003	2.58 (1.71–3.88)	<0.001	2.65 (1.8–3.9)	<0.001
No ICU care	0.31 (0.12–0.81)	0.018	NA	NA	NA	NA
**Prevalent variant**						
Ancestral	ref		ref		ref	
Alpha/Beta	1.44 (0.65–3.21)	0.370	0.76 (0.27–2.16)	0.605	0.67 (0.3–1.52)	0.341
Delta	1.05 (0.52–2.12)	0.893	0.92 (0.35–2.4)	0.870	0.83 (0.43–1.59)	0.572
Omicron	1.27 (0.6–2.69)	0.534	0.88 (0.37–2.11)	0.775	0.87 (0.44–1.72)	0.687
**Treatment**						
Any supplemental oxygen in L/min	NA	NA	0.15 (0.03–0.72)	0.018	0.16 (0.03–0.8)	0.025
Remdesivir	1.43 (0.96–2.13)	0.077	1.15 (0.74–1.78)	0.532	0.24 (0.08–0.76)	0.015
Dexamethasone	0.83 (0.55–1.26)	0.387	0.57 (0.22–1.46)	0.239	0.99 (0.64–1.53)	0.953
**Variant with treatment interaction**						
Ancestral (yes)§	5.63 (2.68–11.81)	<0.001	ref		ref	
Alpha/Beta (yes)	2.83 (1.25–6.41)	0.012	1.93 (0.48–7.84)	0.357	6.96 (1.54–31.43)	0.012
Delta (yes)	2.73 (1.56–4.77)	<0.001	1.41 (0.44–4.56)	0.563	4.13 (1.17–14.58)	0.028
Omicron (yes)	2.84 (1.56–5.16)	0.001	2.4 (0.77–7.52)	0.133	8.89 (2.46–32.13)	0.001

aRR – adjusted risk ratio, CI – confidence interval, ICU – intensive care unit, MEWS – modified early warning score, NA – not applicable, ref – reference

*Model 1: variant with oxygen interaction.

†Model 2: variant with dexamethasone interaction.

‡Model 3: variant with remdesivir interaction.

§Oxygen requirement <15L/min is the reference for model 1.

We found an 85% (aRR = 0.15; 95% CI = 0.03–0.72) reduction in case fatality among participants who received any supplemental oxygen (L/min) compared to those who did not (**Table 4**, model 2). However, across all variants (**Table 4**, model 1), those who required ≥15 L/min oxygen had a significant increase in case fatality compared to those who required <15 L/min oxygen (aRR = 5.63; 95% CI = 2.68–11.81 for ancestral variant, aRR = 2.83; 95% CI = 1.25–6.41 for alpha/beta variant, aRR = 2.73; 95% CI = 1.56–4.77 for delta variant, aRR = 2.84; 95% CI = 1.56–5.16 for omicron variant). Although dexamethasone reduced the case fatality by 43% (aRR = 0.57; 95% CI = 0.22–1.46), the result did not reach statistical significance and the interaction by variant was null (**Table 4**, model 2). Regarding the remdesivir, the interaction term between prevalent variant and remdesivir suggested that the therapy was associated with an higher case fatality during the alpha/beta (aRR = 6.96; 95% CI = 1.54–31.43), delta (aRR = 4.13; 95% CI = 1.17–14.58) and omicron waves (aRR = 8.89; 95% CI = 2.46–32.13) compared to the ancestral wave (**Table 4**, model 3).

The median length of stay among COVID-19 patients decreased by 54% (aRR = 0.55; 95% CI = 0.36–0.84) during the delta wave and by 48% (aRR = 0.49; 95% CI = 0.33–0.74) during the omicron wave compared to the ancestral wave (**Table 5**, model 1). We observed a significant association between remdesivir and median hospital stays (aRR = 1.19; 95% CI = 1.01–1.4). Oxygen therapy and dexamethasone had no impact on length of stay across all variants (**Table 5**, models 1 and 2).

**Table 5:** Factors associated with length of hospital stay in alpha/beta, delta, and omicron variant compared with ancestral variant in COVID-19 patients

	Model 1*		Model 2†		Model 3‡	
	**aRR (95% CI)**	***P*-value**	**aRR (95% CI)**	***P*-value**	**aRR (95% CI)**	***P*-value**	
**Characteristic**							
Male	1.05 (0.91–1.21)	0.491	1.06 (0.92–1.22)	0.427	1.04 (0.9–1.2)	0.557	
Age	1.01 (1–1.01)	0.013	1.01 (1–1.01)	0.012	1.01 (1–1.01)	0.014	
Hypertension	0.95 (0.82–1.1)	0.504	0.95 (0.82–1.09)	0.466	0.94 (0.82–1.09)	0.439	
**Disease severity**							
Moderate	ref		ref		ref		
Severe	1.24 (0.84–1.85)	0.281	1.25 (0.83–1.87)	0.279	1.24 (0.83–1.84)	0.298	
Critical	1.19 (0.77–1.82)	0.435	1.18 (0.76–1.82)	0.472	1.16 (0.75–1.78)	0.511	
MEWS≥4	0.88 (0.76–1.02)	0.098	0.88 (0.76–1.02)	0.096	0.88 (0.76–1.02)	0.094	
**Prevalent variant**							
Ancestral	ref		ref		ref		
Alpha/beta	0.82 (0.58–1.16)	0.262	0.78 (0.58–1.07)	0.121	0.83 (0.64–1.08)	0.161	
Delta	0.55 (0.36–0.84)	0.005	0.59 (0.44–0.78)	<0.001	0.64 (0.51–0.79)	<0.001	
Omicron	0.49 (0.33–0.74)	0.001	0.51 (0.37–0.69)	<0.001	0.52 (0.41–0.67)	<0.001	
**Treatment**							
Any supplemental oxygen in L/min	1.01 (0.65–1.58)	0.949	1.14 (0.76–1.72)	0.522	1.15 (0.77–1.72)	0.490	
Remdesivir	1.18 (1–1.39)	0.051	1.19 (1.01–1.4)	0.038	1.00 (0.73–1.39)	0.977	
Dexamethasone	1.02 (0.86–1.2)	0.859	0.86 (0.66–1.13)	0.275	1.03 (0.87–1.22)	0.752	
**Variant with treatment interaction**							
Ancestral (yes)	ref		ref		ref		
Alpha/Beta (yes)	0.96 (0.62–1.5)	0.867	1.08 (0.7–1.68)	0.720	0.97 (0.6–1.57)	0.902	
Delta (yes)	1.32 (0.83–2.08)	0.238	1.31 (0.91–1.9)	0.149	1.25 (0.85–1.83)	0.254	
Omicron (yes)	1.22 (0.75–1.98)	0.433	1.26 (0.81–1.98)	0.303	1.44 (0.85–2.45)	0.173	

aRR – adjusted risk ratio, CI – confidence interval, ICU – intensive care unit, MEWS – modified early warning score, NA – not applicable, ref – reference

*Model 1: variant with oxygen interaction.

†Model 2: variant with dexamethasone interaction.

‡Model 3: variant with remdesivir interaction.

A subgroup analysis involving moderate to severe COVID-19 cases (n = 473) revealed that dexamethasone reduced fatality by 78% (aRR = 0.22; 95% CI = 0.06–0.84) in this population after adjusting for age, sex, hospital stay, ICU requirement, and prevalent variant in the multivariable robust Poisson regression models. However, we saw no significant association between fatality and remdesivir in the moderate to severe COVID-19 subgroup (aRR = 0.59; 95% CI = 0.20–1.75) (Tables S3–4 in the **Online Supplementary Document**).

## DISCUSSION

In this multicentre observational study, we investigated the differences in the clinical presentation, disease progression, and impact of widespread usage of oxygen, remdesivir, and dexamethasone across four waves (ancestral (November 2020 to March 2021), alpha/beta (March to May 2021), delta (July to September 2021), and omicron (January to February 2022)) of COVID-19 in Dhaka, Bangladesh. We highlight five important observations: hospitalised patients had comparable case fatality rates across waves; oxygen therapy was associated with an 85% reduction in the case fatality rate, but patients requiring ≥15 L/min faced significantly higher fatality compared to those needing <15 L/min, consistent across all variants; remdesivir was associated with a higher case fatality rate during the alpha/beta, delta, and omicron waves compared to the ancestral wave; dexamethasone did not impact death rates across variants but reduced case fatality rates by 78% in the moderate to severe COVID-19 subgroup; and vaccination was associated with a significant reduction in case fatality rates compared to the non-vaccinated group.

A study from South Africa that compared outcomes of patients across four consecutive COVID-19 waves found a significantly reduced risk of death in the omicron wave compared to the three previous waves [27]. Another study conducted between November 2021 and January 2022 in England reported that the risk of hospital admission or death with omicron was approximately one-third that of delta, after adjusting for patient characteristics and vaccination status [28]. However, we observed similar risks of death during the delta and omicron waves. This observed difference might be due to inclusion of mild or asymptomatic omicron cases in these studies, such as in the study from the South African hospital [27], where only 322 (7%) among the 5144 cases required hospitalisation and 45 (0.9%) were diagnosed as severe COVID-19. We found that, among 226 hospitalised omicron cases, 26% had severe COVID-19 and 33% had critical COVID-19, justifying the observed fatality rate comparable to pre-omicron waves.

Apart from death, we observed milder clinical symptoms during the omicron-dominated wave, which is likely due to high exposure to prior infection and vaccination coverage in this setting [29,30]. The combination of natural infection and vaccination, known as hybrid immunity, may offer enhanced protection against new COVID-19 infections and severe cases when compared to either natural infection or vaccination alone. This underscores the significance of vaccination, even for individuals with a past history of COVID-19 [31]. We found that during the omicron wave, 100% of patients received at least one dose of COVID-19 vaccine and 87% received two doses. It has been estimated that, during our study period, roughly 50% of the total Bangladeshi population received at least one dose and approximately 25% received two doses of COVID-19 vaccines [32]. We noted a 37% decrease in in-hospital case fatality among individuals who received the COVID-19 vaccine compared to those who were not vaccinated. The established efficacy of COVID-19 vaccines extends to lowering mortality, mitigating disease severity, and reducing the incidence of new infections. Vaccination has emerged as a key tool in the worldwide efforts to manage the virus’ spread and alleviate the pandemic’s impact on public health [33].

Since the beginning of the pandemic, oxygen has been one of the essential treatments for the optimal management of hypoxemic COVID-19 patients and has saved many lives, as demonstrated in our previous study [20]. The WHO recommended average oxygen flow rate for hypoxemic COVID-19 patients to be 10 L/min, particularly for those not requiring intensive care support [34]. We found that once the oxygen requirement rose to ≥15 L/min, the case fatality increased severalfold across all variants. The aRR of 5.63 among patients requiring higher oxygen (≥15 L/min) during the ancestral wave translates to a 463% increased risk compared to lower oxygen recipients (<15 L/min). Similarly, we observed a 183%, 173%, and 184% increased risk of death during the alpha/beta, delta, and omicron waves, respectively. Previous studies have provided insight into the clinical course and mortality of severe COVID-19 patients with high oxygen demand requiring invasive or non-invasive ventilation [35–37].

We found that remdesivir-treated patients experienced higher case fatality rates during alpha/beta and omicron waves compared to the ancestral wave. We must mention here that remdesivir therapy was not a routine practice in the study hospitals. Physicians preferred remdesivir in severe or critical cases only if participants could purchase the drug. Thus, the association between remdesivir and the case fatality rate might indicate selection bias. A systematic review evaluating the effectiveness of remdesivir among 11 218 participants in high-resource settings concluded that there is minimal to no impact of remdesivir on the mortality of hospitalised patients compared to standard care or placebo [38]. Another review by Yasir et al. observed that patients with mild or moderate COVID-19 might benefit from remdesivir [39]. The Infectious Diseases Society of America has also recommended the use of remdesivir in mild to moderate cases with a high risk of disease progression [40]. We found that 32% of our participants had moderate disease, while 68% had severe to critical disease, indicating that in low-resource settings, individuals seek care and are hospitalised with advanced disease. Thus, the unfavourable impact of remdesivir is not unexpected in this population. However, to our knowledge, no clinical study investigated the differential effect of remdesivir between SARS-CoV2 variants.

We observed shorter hospital stay during the delta and omicron wave compared to the ancestral wave. This reflects a better understanding of the disease and a tendency for earlier discharge during the subsequent waves. Evidence from meta-analyses and clinical trials suggests that dexamethasone reduces mortality in hospitalised COVID-19 patients on oxygen therapy or mechanical ventilation [20,41]. Based on the RECOVERY trial results [17], dexamethasone was the standard of care for severe or critical COVID-19 cases requiring oxygen therapy in our study hospitals. We did not observe its clinically significant impact in our entire cohort. Nevertheless, dexamethasone reduced the case fatality rate by 78% among the moderate to severe COVID-19 subgroup. Thus, the inclusion of more critical COVID-19 cases and several factors, such as the lack of medical resources or advanced ICU settings; the impact of age; and concomitant administration of various treatments might be responsible for the apparent lack of dexamethasone effect in the entire cohort.

This study has some limitations. First, its observational nature suggests that the outcomes could be affected by potential unmeasured confounders. For example, confounding by indication is a potential limitation because the decision to administer treatments, such as oxygen ≥15 L/min and dexamethasone in particular, was influenced by the severity of the illness. Furthermore, the use of remdesivir was contingent on individuals' purchasing capabilities, thereby influencing clinicians' treatment decisions, which could potentially impact the remote reduction of comparability. Nevertheless, we addressed potential confounders such as the severity of COVID-19 and MEWS by employing multivariable robust Poisson regression models in our analysis. Similarly, the apparent lack of an effect of dexamethasone in our study may be attributed to methodological limitations. Furthermore, patients who required ≥15 L/min of oxygen were considered critically ill, necessitating oxygen delivery through advanced devices such as HFNC or mechanical ventilation. However, only 21% of the 109 patients needing higher oxygen (≥15 L/min) received HFNC or mechanical vantilation, highlighting resource constraints in LMICs.

Second, the retrospective data collection during the pre- and post-delta wave could have introduced selection bias, especially concerning fatal cases. Consequently, the case fatality rates may differ between the pre- and post-delta waves and the delta wave. Third, the results of this single-site study may not be extrapolated to other countries or advanced settings. Fourth, we collected data from five different hospitals and, although they followed the national COVID-19 guideline for case management, we observed substantial variability regarding oxygen, remdesivir, dexamethasone, and antibiotic therapy across the facilities. Therefore, differences in treatment practices between hospitals could potentially confound the association between variants and treatment outcomes. Finally, our study has covered the period both before and after the initiation of COVID-19 vaccination programs. Immunisation might have contributed to substantial variations in disease severity and clinical management.

Future research should prioritise the study of new variants and their impact on disease severity, mortality, and vaccine efficacy. Randomised controlled trials and real-world studies are crucial in low-resource settings to assess the clinical outcomes and efficacy of different treatment options for individuals infected with forthcoming SARS CoV-2 variants, aiming to identify the best practices and guidelines for clinical management.

## CONCLUSIONS

We observed that the 30-day case fatality rates remained comparable across different SARS-CoV-2 variants. However, we found notable differences in symptomatology, particularly during the omicron wave. Importantly, oxygen therapy (L/min) showed an 85% reduction in case fatality rates, emphasising its critical role in patient outcomes. The higher risk of case fatality among individuals requiring ≥15 L/min oxygen across all variants underscores the need for vigilant care for such patients. We did not find a significant impact of remdesivir on case fatality rates, emphasising the challenges of managing progressively deteriorating severe or critical COVID-19 patients in resource-limited settings like Bangladesh. Our findings reiterate the significance of sustained vaccination efforts and the need for clinical trials of newer antivirals in the ongoing battle against COVID-19. They also provide valuable information for public health strategies, clinical decision-making, and further research in navigating the consequences of future SARS-CoV-2 mutations.

## Additional material


Online Supplementary Document.
