# Supplementary material for: Clinical characterisation, treatment outcomes, and case fatality risk of patients with different SARS-CoV-2 variants in Bangladesh
Source: J Glob Health. 2024 Jun 21;14:05009. doi: 10.7189/jogh.14.05009 (PMC11191382; doi:10.7189/jogh.14.05009)
Supplement: Online Supplementary Document. [file jogh-14-05009-s001.pdf]

ONLINE SUPPLEMENTARY DOCUMENT

Title: Clinical characterization, treatment outcomes, and case fatality risk of patients with different SARS-CoV-2 variants in Bangladesh

Farzana Afroze[1], Mst. Noorjahan Begum[2], Tahmeed Ahmed[1], Dr Shams El Arifeen[3], Mohammed Ziaur Rahman[4], Ahmed Ehsanur Rahman[3], Mustafa Mahfuz[1], Md. Farhad Kabir[1], Ahmedul Kabir[5], Md. Robed Amin[6], Mohammad Shehab Uddin[7], Muhammad Asaduzzaman[8], M A Hasnat[9], Md. Khairul Islam[10], Md Mohiuddin Sharif[10], Rezaul Hossain[1, 11], Yasmin Jahan[12], Md. Mustafizur Rahman[2†], Mohammod Jobayer Chisti[1†]

†Joint senior authorship.

Table S1. Actual risk of death in hospitalized COVID-19 patients, in Dhaka, Bangladesh.

	**Fatal cases n=111**	**Survivors n=755**	**Absolute Risk**	**RR (95% CI)**	***P*-value**
**Sex**					
Female	41 (36.9)	405 (47.4)	9.19	Ref.	
Male	70 (63.1)	450 (52.6)	13.46	1.46 (1.02, 2.11)	0.038
**Age Category**					
18 to 40 years	4 (3.6)	186 (21.8)	2.11	Ref.	
41 to 60 years	28 (25.2)	412 (48.2)	6.36	3.02 (1.07, 8.50)	0.036
Above 60 years	79 (71.2)	257 (30.1)	23.51	11.17 (4.15, 30.03)	<0.001
**Disease severity**					
Moderate	7 (6.3)	299 (35.0)	2.29	Ref.	
Severe	13 (11.7)	154 (18.0)	7.78	3.40 (1.38, 8.37)	0.008
Critical	91 (82.0)	402 (47.0)	18.46	8.07 (3.79, 17.2)	<0.001
MEWS ≥4	71 (64.0)	315 (36.8)	18.39	2.67 (1.85, 3.84)	<0.001
**Prevalent Variant**					
Ancestral	19 (17.1)	180 (21.1)	9.55	Ref.	
Alpha/Beta	23 (20.7)	121 (14.2)	15.97	1.67 (0.95, 2.95)	0.076
Delta	45 (40.5)	354 (41.4)	11.28	1.18 (0.71, 1.96)	0.521
Omicron	24 (21.6)	200 (23.4)	10.71	1.12 (0.63, 1.99)	0.692
**Vaccine against COVID-19**					
Vaccinated	18 (19.0)	219 (28.6)	7.59	Ref.	
Not vaccinated	77 (81.0)	546 (71.4)	12.36	1.63 (1.0, 2.66)	0.052

Data are n (%), RR, risk ratio; CI, confidence interval; MEWS, Modified Early Warning Score.

Table S2. Impact of COVID-19 vaccine and risk of case fatality among hospitalized COVID-19 patients in Dhaka, Bangladesh.

**Characteristics**	**aRR (95% CI)**	***P*-value**
COVID-19 vaccine	0.63 (0.40, 0.99)	0.048
Age Category		
Above 60 years	Ref.	
18 to 40 years	0.12 (0.04, 0.32)	<0.001
41-60 years	0.27 (0.17, 0.43)	<0.001
Hospital stay (in days)	0.86 (0.81, 0.91)	<0.001
ICU not required	0.29 (0.16, 0.52)	<0.001
SpO2 level	0.97 (0.96, 0.98)	<0.001

aRR, adjusted risk ratio; CI, confidence interval.

Table S3. Impact of dexamethasone and risk of case fatality in ancestral, alpha/beta, and omicron variants compared with delta among moderate to severe COVID-19 patients.

**Characteristics**	**aRR (95% CI)**	***P*-value**
Not received dexamethasone	Ref.	
Received dexamethasone	0.22 (0.06, 0.84)	0.026
Age		
Above 40 Year	Ref.	
18 to 40 Years	0.33 (0.04, 2.57)	0.287
Female	Ref.	
Male	0.76 (0.29, 1.94)	0.562
Hospital stay (days)	1.02 (0.97, 1.08)	0.390
Required ICU care	Ref.	
Not required ICU care	0.05 (0.02, 0.13)	<0.001
Prevalent variant		
Delta	Ref.	
Ancestral	0.37 (0.16, 0.88)	0.024
Alpha/Beta	0.79 (0.19, 3.26)	0.746
Omicron	0.21 (0.06, 0.76)	0.018

aRR, adjusted risk ratio; CI, confidence interval.

Table S4. Impact of remdesivir and risk of case fatality in ancestral, alpha/beta, and omicron variants compared with delta among moderate to severe COVID-19 patients.

**Characteristics**	**aRR (95% CI)**	***P*-value**
Not received remdesivir	Ref.	
Received remdesivir	0.59 (0.20, 1.75)	0.345
Age		
Above 40 Year	Ref.	
18 to 40 Years	0.35 (0.04, 2.74)	0.317
Female	Ref.	
Male	1.02 (0.44, 2.37)	0.964
Hospital stay	1.01 (0.95, 1.07)	0.703
Required ICU care	Ref.	
Not required ICU care	0.07 (0.03, 0.18)	<0.001
Prevalent Variant		
Delta	Ref.	
Ancestral	0.44 (0.17, 1.14)	0.090
Alpha/Beta	0.77 (0.20, 2.93)	0.703
Omicron	0.24 (0.06, 0.88)	0.031

aRR, adjusted risk ratio; CI, confidence interval.
